# Antidepressants in postural orthostatic tachycardia syndrome (POTS): A scoping review

**DOI:** 10.1007/s10072-026-09542-y

**Published:** 2026-11-11

**Authors:** Svetlana Blitshteyn, Angela M. Reiersen, Leonard B. Weinstock, Traci J. Speed, Glenn J. Treisman

**Affiliations:** 1 Department of Neurology, Jacobs School of Medicine and Biomedical Sciences, University at Buffalo, 955 Main Street, Buffalo, NY 14203 USA; 2 Dysautonomia Clinic, Williamsville, NY USA; 3 Department of Psychiatry, Washington University in St. Louis School of Medicine, St. Louis, MO USA; 4 Gastrointestinal Alliance, Washington University School of Medicine, St. Louis, MO USA; 5 Department of Psychiatry, Johns Hopkins University School of Medicine, Baltimore, MD USA

**Keywords:** Postural orthostatic tachycardia syndrome, Antidepressants, SSRIs, Therapies, Evidence-based medicine

## Abstract

**Background:**

Postural orthostatic tachycardia syndrome (POTS) is one of the most common autonomic disorders with increasing prevalence and incidence after COVID-19 pandemic. Despite standard treatment of POTS consisting of nonpharmacologic and pharmacologic therapies that decrease heart rate and increase blood pressure and plasma volume, antidepressant medications are widely utilized in patients with POTS.

**Methods:**

We conducted a scoping review of literature to examine the published studies on the use of antidepressants and their efficacy in patients with POTS.

**Results:**

We found only 2 studies, one retrospective chart review involving bupropion and second study of a randomized placebo-controlled crossover trial of a single dose of sertraline 50 mg. Both studies showed no benefits of these medications on orthostatic vital signs or orthostatic symptoms. A third study involving retrospective chart review did not examine medication efficacy, but found that nearly half of 120 patients with POTS were taking some type of antidepressant or antianxiety medication while only 15.7% were taking cardiovascular medications for treatment of POTS.

**Conclusions:**

Based on very limited available data, we conclude that antidepressants show no efficacy for treatment of POTS despite being widely utilized. Education of healthcare professionals regarding proper diagnosis and appropriate treatment of POTS is essential to instituting the right treatment and avoiding inappropriate use of antidepressants in this patient population.

## Introduction

Antidepressants, many of which are serotonin, norepinephrine and/or dopamine reuptake inhibitors, are widely prescribed in primary care for conditions other than depression and anxiety, such as fibromyalgia, neuropathic pain, irritable bowel syndrome and dysautonomia, including postural orthostatic tachycardia syndrome (POTS). Concerns have been raised about potential over-prescribing of selective serotonin reuptake inhibitors (SSRIs) in the United States [1]. Despite their widespread use, there is a significant paucity of data on the use of antidepressants in patients with POTS and whether these are effective for POTS.

## Methods

We conducted a scoping review in search of studies examining the efficacy of antidepressants in patients with POTS using PubMed. Inclusion criteria were: (1) either retrospective or prospective studies (2) patients with POTS. We excluded case reports, case series, opinion/perspectives and review articles. We also excluded studies on antidepressants in other autonomic disorders, such as vasovagal syncope and orthostatic hypotension.

## Results

We identified a total of 2 studies evaluating the use of antidepressants in patients with POTS (Table 1). One study was a non-randomized retrospective chart review on 47 patients with POTS that found no evidence of a statistically significant improvement in orthostatic heart rate with the use of bupropion - a norepinephrine and dopamine reuptake inhibitor. Patients with POTS did report a decrease in syncope frequency [2]. A second study, which was a randomized, placebo-controlled, crossover trial of 39 patients with POTS, found no reduction in standing heart rate and no improvement in symptoms of orthostatic intolerance with a one-time dose of sertraline 50 mg vs. placebo at 4 h of standing [3]. In fact, orthostatic intolerance symptoms actually worsened after sertraline compared to placebo [3]. A third study provided a retrospective analysis on the use of antidepressant and antianxiety medications in 120 patients with POTS demonstrating a significant increase in the use of antidepressant medications over a decade, and especially following COVID-19 pandemic, with 48.3% taking antidepressants or antianxiety medication while percentage of patients treated with cardiovascular medications was very low at 15.7% [4].

**Table 1:** Antidepressants in patients with POTS: summary of the available data

Study/Medication	Study design	Findings
Vyas et al. 2020/ Bupropion	Retrospective chart review of 47 patients	No improvement in orthostatic vital signs; improvement in self-reported syncope frequency
Mar et al. 2014/ Sertraline 50 mg one dose	Randomized placebo-controlled crossover study of 39 patients	No reduction in standing heart rate or improvement in symptoms; seated blood pressure increased modestly and symptoms were worse at 4 h versus placebo after a single dose of sertraline
Shrestha et al. 2026/ Antidepressants/antianxiety medications: 15.6% SSRIs 8.9% SNRIs 7.5% “other”	Retrospective chart review of 120 patients	48.3% of patients with POTS were taking antidepressant/antianxiety medication in 2025 vs. 15.7% of patients taking heart rate medications.

There were no other published studies identified on the use of any other antidepressants in patients with POTS.

## Discussion

In this scoping review, we found only 2 studies assessing the efficacy of antidepressants in patients with POTS, both of which failed to demonstrate any positive effect of these medications on orthostatic tachycardia or symptoms of orthostatic intolerance [2, 3]. Despite their widespread use, no evidence exists to support the use of antidepressants for treatment of orthostatic symptoms and heart rate elevation in patients with POTS.

A third study by Shrestha et al. demonstrated that 32% of patients referred for a tilt table test and 48.3% of patients with POTS were taking antidepressant or antianxiety medication, highlighting a concerning trend: nearly half of patients with POTS are prescribed psychotropics despite no randomized trials of these medications in patients with POTS [4]. It is unclear how many of these individuals actually met criteria for a psychiatric diagnosis. By the time patients get a tilt table test or see a specialist, nearly a half of POTS patients have already been prescribed SSRIs or SNRIs by a healthcare professional [4]. We believe that in a large subset of patients, these medications may have been prescribed due to POTS symptoms being misattributed to symptoms of depression or anxiety. Furthermore, clinical observation and retrospective data from Shrestha et al. show that many patients have not been trialed beta blockers, midodrine, ivabradine, fludrocortisone or pyridostigmine – evidence-supported medications used as first-line treatment for POTS and other autonomic disorders [4].

Although neurologic manifestations of POTS are numerous, POTS is not caused by generalized anxiety disorder, panic disorder, major depression, somatization or functional neurologic disorders [5]. Antidepressants have significant side effects, including long-term adverse events and dependance potential, which should preclude their liberal use and overuse in patients with chronic medical conditions. Antidepressants can cause tachycardia, sleep disturbance and gastrointestinal side effects – symptoms that are already highly prevalent in patients with POTS [6].

Based on the available studies, we conclude that there is no evidence to date that antidepressants should be used as treatment for POTS. If there is comorbid generalized anxiety disorder, major depression or another psychiatric disorder in a patient with POTS, then a trial of SSRIs, SNRIs, low-dose tricyclic antidepressants (TCAs) or bupropion may be appropriate to treat these psychiatric comorbidities. Bupropion may be particularly attractive given its efficacy in treatment of fatigue, which is one of the most common and disabling symptoms of POTS [7]. SNRIs and TCAs may also benefit patients with comorbid chronic pain, especially with neuropathic pain from small fiber neuropathy, which is highly prevalent in patients with POTS [8].

Overprescribing/misprescribing antidepressants for patients with POTS is concerning when it is used inappropriately for treatment of POTS symptoms in patients without confirmed psychiatric disorders and may warrant deprescribing considerations [9]. Because POTS has been reported as a major post-acute long-term complication of SARS-CoV-2 infection, it is especially important to properly diagnose and treat POTS. Interestingly, a recent study identified the use of antidepressants as a risk factor for development of Long COVID and post-COVID POTS in children and adolescents who were taking these medications before COVID-19 infection [10] suggesting that both their use and indications for their use deserve consideration and further studies.

Limitations of this review include an extremely limited number of the available studies. Additionally, we did not search for studies on antidepressants in patients with other autonomic disorders, besides POTS. Finally, the use of antidepressant and antianxiety medications may be beneficial in patients with POTS if accompanied by confirmed psychiatric comorbidities, such as generalized anxiety disorder, major depression or panic disorder, and these comorbidities may warrant treatment with these medications.

## Conclusion

There is no evidence to date that antidepressants are effective for treatment of POTS symptoms despite their widespread use in clinical practice. Inappropriate treatment with antidepressants instead of evidence-based treatment with blood volume expansion, compression garments, beta blockers, ivabradine, midodrine and other targeted treatment for autonomic dysfunction, may lead to negative patient outcomes, stigma associated with psychiatric treatment and poor functional state when POTS symptoms remain untreated. Ultimately, medical education and training of primary care physicians, neurologists, cardiologists and other specialists on how to properly diagnose and treat patients with POTS and other common autonomic disorders is the greatest challenge but is an essential step toward patients receiving appropriate, evidence-based treatment. While due to a lack of evidence antidepressants should not be used as treatment of POTS symptoms, future clinical trials are needed to develop FDA-approved therapies for POTS, determine if patients with POTS and psychiatric comorbidities benefit from antidepressants and track deprescribing outcomes.
